# Individual health behaviours to combat the COVID‐19 pandemic: lessons from HIV socio‐behavioural science

**DOI:** 10.1002/jia2.25771

**Published:** 2021-08-02

**Authors:** Jessica E Haberer, Ariane van der Straten, Steven A Safren, Mallory O Johnson, K Rivet Amico, Carlos del Rio, Michele Andrasik, Ira B Wilson, Jane M Simoni

**Affiliations:** ^1^ Center for Global Health Massachusetts General Hospital Boston MA USA; ^2^ Department of Medicine Harvard Medical School Boston MA USA; ^3^ ASTRA Consulting Kensington CA USA; ^4^ Department of Medicine University of California San Francisco San Francisco CA USA; ^5^ Department of Psychology and Center for HIV and Research in Mental Health University of Miami FL USA; ^6^ Health Behavior & Health Education University of Michigan School of Nursing Ann Arbor MI USA; ^7^ Department of Global Health Rollins School of Public Health Atlanta GA USA; ^8^ Division of Infectious Diseases Emory University School of Medicine Atlanta GA USA; ^9^ Vaccine and Infectious Disease Division, Fred Hutchinson Cancer Research Center Seattle WA USA; ^10^ Department of Health Services, Policy and Practice Brown University Providence RI USA; ^11^ Departments of Psychology and Global Health University of Washington Seattle WA USA

**Keywords:** HIV, COVID‐19, social science, behavioural science, public health, vaccine

## Abstract

**Introduction:**

COVID‐19 parallels HIV in many ways. Socio‐behavioural science has been critical in elucidating the context and factors surrounding individual levels of engagement with known effective prevention and treatment tools for HIV, thus offering important lessons for ongoing efforts to combat the COVID‐19 pandemic.

**Discussion:**

Non‐adherence to effective disease mitigation strategies (e.g. condoms for HIV and masks for COVID‐19) can be attributed in part to prioritizing comfort, convenience and individual autonomy over public health. Importantly, misinformation can fuel denialism and conspiracies that discredit scientific knowledge and motivate nonadherence. These preferences and the extent to which individuals can act on their preferences may be constrained by the structures and culture in which they live. Both HIV and COVID‐19 have been politicized and influenced by evolving recommendations from scientists, clinicians, policymakers and politically motivated organizations. While vaccines are vital for ending both pandemics, their impact will depend on availability and uptake. Four decades of experience with the HIV epidemic have shown that information alone is insufficient to overcome these challenges; interventions must address the underlying, often complex factors that influence human behaviour. This article builds from socio‐behavioural science theory and describes practical and successful approaches to enable and support adherence to prevention and treatment strategies, including vaccine adoption. Key methods include reframing tools to enhance motivation, promoting centralized sources of trusted information, strategic development and messaging with and within key populations (e.g. through social media) and appealing to self‐empowerment, altruism and informed decision making. Orchestrated evidence‐based activism is needed to overcome manipulative politicization, while consistent transparent messaging around scientific discoveries and clinical recommendations are critical for public acceptance and support. Ultimately, the effectiveness of COVID‐19 vaccines will depend on our ability to engender trust in the communities most affected.

**Conclusions:**

Many lessons learned from socio‐behavioural science in the HIV pandemic are applicable to the COVID‐19 pandemic. Individual behaviour must be understood within its interpersonal and societal context to address the current barriers to adherence to disease‐mitigating strategies and promote an effective response to the COVID‐19 pandemic, which is likely to be endured for the foreseeable future.

## 1 Introduction

Effectively combating the COVID‐19 pandemic requires an arsenal of biomedical prevention and treatment interventions. In concert with these approaches, non‐pharmacologic mitigation strategies will necessitate changes in human behaviour, without which successful control of the virus will be impossible. In this respect, the HIV pandemic provides important lessons for the public response to COVID‐19.

These lessons stem from important parallels between the two pandemics. First, both HIV and SARS‐CoV‐2 (the virus that causes COVID‐19 disease) are infectious pathogens that require individual facing and social and structural interventions at local and global levels. Both pandemics highlight health disparities in society, disproportionately affecting poor and marginalized populations, as well as heightened transmission in some niche settings [1, 2, 3, 4]. Reducing transmission for each relies at least in part on individual‐level behaviours (e.g. taking antiretroviral medication for treatment or prevention, HIV testing, syringe exchange and condom use for HIV; physical distancing and wearing a mask for COVID‐19), and these behaviours must be ongoing (e.g. daily or during sex for HIV; whenever in public spaces for COVID‐19) for the foreseeable future. With both HIV and COVID‐19, tension exists between these mitigation strategies that bolster individual and community protection versus personal desires (e.g. sexual intimacy with HIV; social gatherings and physical contact with COVID‐19). Adoption of and persistence in these behaviours is critical to prevent transmission of both viruses, yet these behaviours must be understood within the structural and cultural context in which individuals operate (e.g. Do women have power to enforce consistent condom use with male sexual partners? Does prevailing local championing of individual civil liberties inhibit mask wearing?).

Differences between HIV and COVID‐19 should be acknowledged as well, including ease and route of transmission [5, 6], accuracy and availability of diagnostic testing [7, 8], disease course [9, 10] including long‐term effects, and the ability to control exposure. While testing positive for both SARS‐CoV‐2 and HIV has been marred with stigma and discrimination, the situation is far worse for the latter [11]. Moreover, oral pre‐exposure prophylaxis (PrEP) has been in an important biomedical HIV prevention tool [12] that lacks a parallel in the COVID‐19 pandemic, and COVID‐19 now has highly effective vaccines that lack a parallel in HIV.

Despite these differences, four decades of the HIV pandemic provide lessons for the public health response in combatting COVID‐19. Socio‐behavioural science, in particular, can help elucidate individuals’ engagement with interventions, as well as identify means to improve the effectiveness of prevention efforts. In this paper, we highlight key barriers and contextual factors that influence individual behaviour, as well as caution against the panacea‐like promise of vaccines. Recognizing that larger social and structural factors have important influences, our goal is to build from the experience and evidence‐based approaches with HIV to advance an effective public response to COVID‐19.

## 2 The role of socio‐behavioural sciences

Socio‐behavioural science theories have addressed many aspects of the HIV epidemic [13] and similarly have relevance for COVID‐19 [14, 15]. While considerations should be tailored for specific prevention or treatment tools or populations, some element of individual‐level execution of a behaviour within an enabling environment are typically required. As a guiding principle, socio‐behavioural science demonstrates information is necessary but insufficient for meaningful behaviour change [16]. Models of health behaviour include variables such as perceived benefits and barriers, self‐efficacy, norms and attitudes, social influences and other social‐cognitive factors. Moreover, models can highlight the importance of addressing social inequities, such as structural racism and human rights violations, that often limit the ability of individuals to enact desired behaviours.

**Figure 1:**
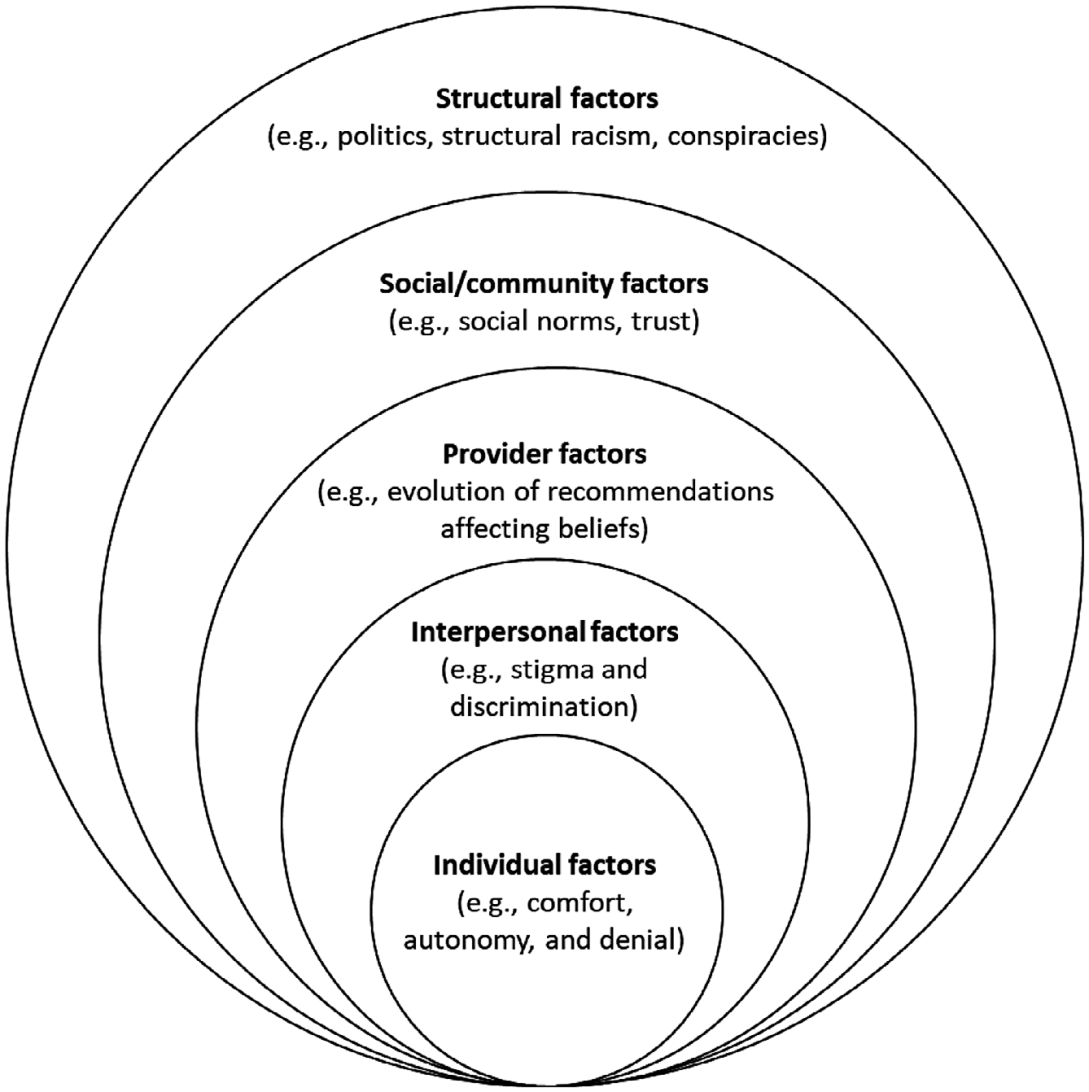
A socio‐ecological model for engagement with interventions against COVID‐19. Adapted from Bronfenbrenner [17].

One model that may guide an effective response to the COVID‐19 pandemic is the socio‐ecological model, which delineates varying levels of factors influencing behaviour (Figure 1) [17]. The model explains how behaviour among individuals with diverse characteristics and beliefs is entwined within interactions between individuals and their particular interpersonal surroundings, as well as societal and structural factors (e.g. a young, wealthy man in a high‐resourced setting vs. a poor, elderly woman in a low‐resourced setting). Interventions should consider this holistic context; targeting individual behaviour in isolation is unlikely to have meaningful impact. Conversely, inadequate access to effective prevention and treatment tools may thwart individual intentions.

**Figure 2:**
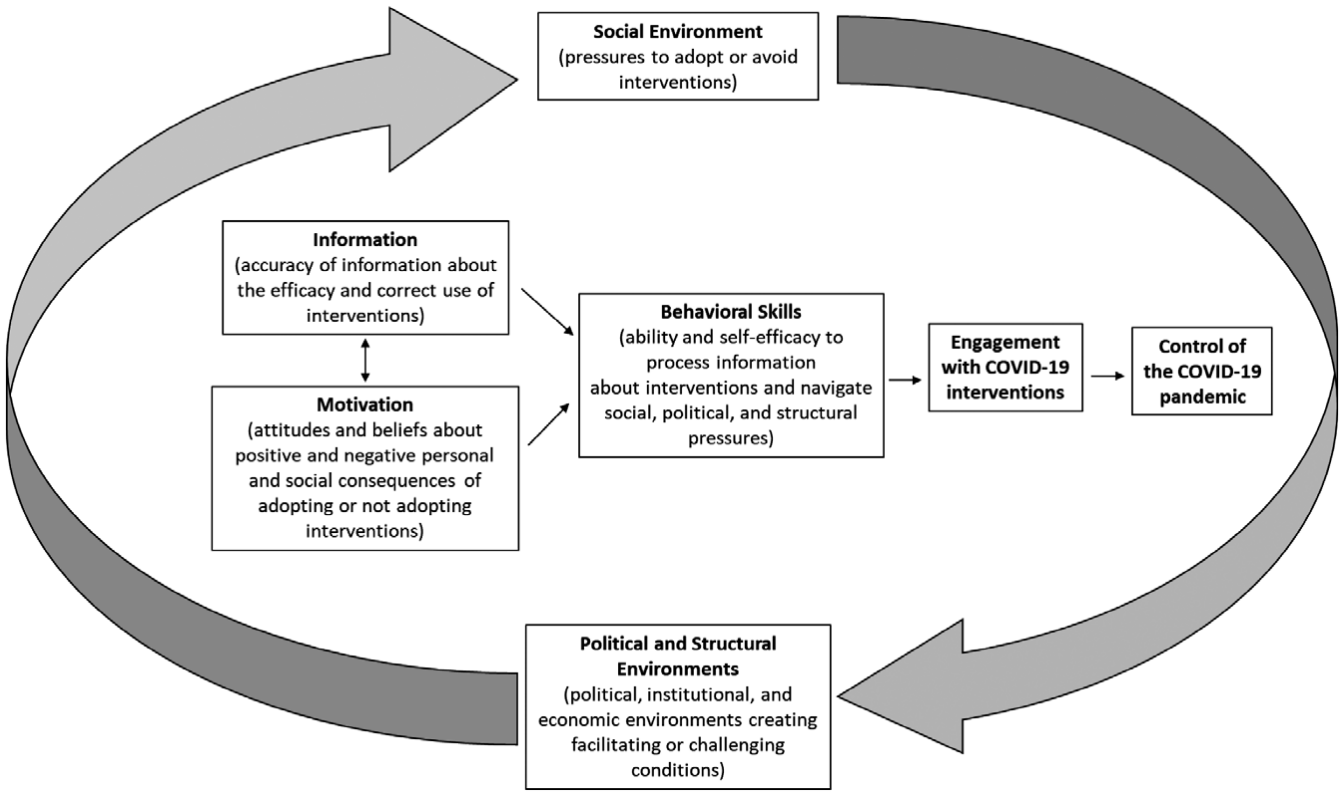
The situated information‐motivation‐behavior model for engagement with interventions against COVID‐19. Adapted from Amico [19].

The Information‐Motivation‐Behavioural Skills (IMB) model consolidates health behaviour change theories into three key variables that influence health behaviours: information, motivation and behavioural skills [18]. Each of these barriers can influence behaviour change and may need to be addressed to enable intervention effectiveness. The situated IMB (sIMB) model [19] considers adoption and execution of behaviours within a context of dynamic influences from internal, personal, social, community and structural factors (Figure 2). While information and motivation (i.e. one’s sense of personal and social consequences, positive and negative, of adopting a preventative behaviour or specifically not adopting it) are important, access to prevention tools and skills in using them across diverse situations is essential [20].

## 3 Engagement with disease mitigation strategies

Mitigation strategies for both HIV and COVID‐19 require acceptance and belief, accompanied by the ability to adhere to recommended use. Adherence is critical to maximizing benefit from HIV prevention and treatment tools and likewise is paramount for combatting COVID‐19. Here, we present three key barriers that limit individual engagement in the response to COVID‐19, particularly as they relate to masks and physical distancing: comfort and convenience, denialism and misinformation and autonomy versus public health.

### 3.1 Immediate comfort and convenience

As with HIV prevention, measures to reduce the spread of SARS‐CoV‐2 necessitate adopting behaviours and implementing them in situations that often create challenges to both comfort and convenience. The primary methods of prevention early in the HIV epidemic were limited to wearing condoms and not sharing drug injection supplies. Comfort and convenience are not trivial concepts. Condoms, for example, can be experienced as uncomfortable and can signal mistrust, introducing stigma into sexual relationships. Importantly, condom use requires cooperation of the insertive partner, which the receptive partner may not always have the power to obtain.

Similarly, mask wearing and physical distancing to prevent SARS‐CoV‐2 is accompanied by a loss of comfort, convenience and normal social intimacy that threaten engrained rituals (e.g. shaking hands) and human communication (e.g. seeing facial expressions). They may also lead to feelings of isolation and can result in stigma in settings where such behaviours are politicized. These constraints limit sustained adherence and ultimately the effectiveness of these interventions. Successful HIV interventions sought to reframe condom use as responsible and even sexy, thus enhancing person‐centric motivation [21]. For example, condoms of different colours and flavours are available that enhance sensation or target different users (e.g. female condoms). HIV prevention interventions have also developed language and techniques to normalize consistent condom use and make putting on condoms a way of enhancing rather than diminishing intimacy [22]. With COVID‐19, we have seen a similar shift in the framing of masks and physical distancing. Masks are being designed with increasing comfort and fashionable styles, and numerous social media platforms have created alternative opportunities for socializing. Efforts to routinize these behaviours and integrate them into social norms may promote adherence.

### 3.2 Denialism and misinformation

Early in the HIV epidemic, stigma and marginalization were fuelled by reactionary forces and conservative religious ideology. Derogatory myths, like the“‘Four H’s’: Homosexuals, Heroin users, Hemophiliacs, and Haitians,” were inappropriately used to lay blame for HIV, and conspiracy theories abounded about the intentional development of HIV as a biological weapon against marginalized subgroups [23]. Indeed, the U.S. President Ronald Reagan did not publicly mention AIDS until many years into the epidemic. Some national COVID‐19 responses and the vast reach of social media have also led to conspiracy theories and misinformation that are fuelling denial of the scope and impact of the pandemic. Numerous heads of state dismissed the virus as a passing, over‐inflated threat and social media fed the idea that it was a hoax. Moreover, responsibility for the response was deflected by calling SARS‐CoV‐2 the “China virus,” as AIDS was once called “Gay‐Related Immune Deficiency, or GRID.” Lack of coordinated public health responses early in both pandemics further exacerbated misinformation and mistrust among the public.

Countering the misinformation that underlies denialism and leads to rationalizing non‐adherence to public health guidelines requires multi‐level intervention. Promoting centralized public sources of trusted information (e.g. the U.S. CDC for HIV; the World Health Organization for COVID‐19) may motivate engagement and normalize adherence to public health interventions. These sources should be combined with locally trusted community organizations and leaders, particularly among marginalized communities. For example HIV prevention has been promoted with African American churches and among community elders in Kenya [24, 25]. Strategic social media use may counteract conspiracy campaigns. Policies to check inaccuracies on social media platforms also may be useful. Direct social norm interventions and public media campaigns that diffuse information through trusted opinion leaders (e.g. Magic Johnson with HIV [26]) within demographic subgroups may additionally promote evidence‐based information and evidence‐informed responses to COVID‐19.

### 3.3 Autonomy and public health

When “authorities” strongly recommend or even mandate behaviour adoption (e.g. mask wearing), the ethical question of autonomy (i.e. the right to make individual choices) may arise. The relative prioritization of autonomy over societal needs varies by culture and individual. With infectious diseases like HIV and COVID‐19, the impact on public health argues for reduced autonomy and behooves public institutions to coordinate activities of individuals for the collective good. Indeed, government‐enforced restrictions have been effective in some settings [27]. However, public health directives may be resisted, often vociferously in libertarian‐leaning settings [28]. Regarding COVID‐19, many have fought regulations to require wearing face masks and social distancing, citing rights to control their own behaviours. With HIV, people have resisted prevention efforts that infringe on personal sexual behaviour (e.g. closing bath houses during outbreaks). Some have also resisted antiretroviral therapy (ART), invoking autonomy in their HIV treatment decisions. Laws developed to regulate these behaviours (e.g. mandating HIV disclosure) failed to address the underlying moral principle of self‐determination and instead have caused harm, including increased stigma and discrimination towards groups associated with HIV and disproportionately affected marginalized communities [29]. Pitting public health against civil liberties raises tensions and mistrust, doing little to address the underlying beliefs.

Messaging that emphasizes individual actions in preventing an infectious disease is an important step in explaining the need for external policies; however, it does not address the fundamental value placed on autonomy. An appeal to voluntarily choose engagement with an intervention is needed. This approach can be seen in the use of self‐empowerment and altruism to prevent spread of HIV. The campaign “U=U,” or “Undetectable equals Untransmittable,” highlights how ART adherence and corresponding viral suppression can extend beyond personal benefit to prevent secondary transmission [30]. For COVID‐19, a similar appeal may help individuals who prize autonomy to see the power of their own choice to wear masks or stay physically distanced to limit transmission and show respect for others. Although stigma, power dynamics and social consequences may inhibit the ability of individual actors, re‐framing prevention efforts to address the moral beliefs of individuals to “do their part,” perhaps in a plea to patriotism, holds promise for increased acceptance and implementation.

## 4 Politicization of the pandemic

Exacerbating these barriers is the way disease mitigation strategies against COVID‐19 have been manipulated for ulterior political motives in some settings, just as HIV prevention has been. Infectious disease and public health experts are often labelled as taking a liberal stance rather than presenting science, while some conservative governmental leaders have cast doubt on the seriousness of COVID‐19 as exaggerated for political gain. Indeed, mask wearing is a political statement in many settings. The linking of COVID‐19 to specific groups, such as immigrants, has also aligned with political values. This politicization has parallels with the HIV pandemic, which has historically been seen by some as affecting marginalized groups who “brought it on themselves” through their sexual behaviour or drug use. At the extreme is a sordid history of criminalization of HIV in many countries, which served to undermine positive public health interventions [29]. With HIV as with COVID‐19, these dynamics resulted in denials of the severity of the crises and delays in national responses and plans for mitigating escalating public health crises. Moreover, anti‐science rhetoric has further polarized the response.

Activism orchestrated by groups affected by HIV and their allies was necessary to raise awareness, normalize experiences and ultimately weaken political divides that blocked progress toward coherent and effective plans for prevention and treatment, including medication access and syringe exchange programs [31]. Indeed, socio‐behavioural scientists have led the call for neglect of these evidence‐based strategies to be considered crimes against humanity [32] and activists have taken action to circumvent policies, including promoting underground syringe exchanges in the 1980s to 1990s [33]. Similar activism has occurred with COVID‐19, including labour strikes for person denied adequate protective equipment [34], and is emerging among groups disproportionately affected by COVID‐19, including teachers, essential workers, the elderly and underrepresented racial and ethnic groups. These voices will need to play a strong role in overcoming the damage caused by manipulative politicization of COVID‐19 that is impeding engagement with effective interventions.

## 5 Evolving recommendations creating confusion and lack of confidence

Although HIV is currently well‐characterized, much controversy initially surrounded its transmissibility and treatment. Best practices to mitigate clinician risk evolved from full body protective gear when treating patients with AIDS in the 1980s to applying universal precautions for all medical procedures. Similarly, improved transmission risk estimates across behaviours and types of contact helped support standardization of prevention recommendations while destigmatizing key populations. Notably, zidovudine, the first antiretroviral medication for treating AIDS in 1986, was highly controversial and perceived by some as poisoning patients, serving only to profit pharmaceutical companies, or even causing AIDS [23]. Development of more effective and tolerable ART regimens and better education and support for the necessity of ART have been needed to reduce mistrust in many communities [35].

Scepticism over biomedical treatment and prevention strategies can be an understandable response, particularly among communities poorly represented in scientific circles and companies disseminating the strategy. This scepticism is further justified given the sordid history of medical experimentation on marginalized populations, ongoing inequities in healthcare and politicization of public health measures [36]. The public health and medical establishment need to acknowledge and address these concerns through approaches to earn trust, and messages that seem changing or contradictory will challenge that trust. For example, early guidelines recommended ART only for advanced HIV disease [37], whereas guidelines later changed to treat all people with HIV as soon as possible, even at diagnosis [38, 39]. Convincing end‐users of the validity of that message, however, took time, as beliefs that ART initiation meant serious health decline had to be updated through public health efforts [40]. Similarly, our understanding of the effectiveness of PrEP evolved as did our understanding of the importance of adherence in achieving high protection with PrEP [41]; this relationship was integrated into normative guidance around its use with common phrases, such as “PrEP works if you take it” [42, 43]. Individual decisions to trust sources of information can be complex; however, credibility may be gained through targeted community engagement and collaborative crafting of messages that speak to their lived experiences, particularly among marginalized communities. Dissemination of these recommendations should be undertaken jointly in partnership with communities. Because the scientific process is inherently dynamic and evidence is likely to be incomplete, at least initially, transparency about what is known and not known is critical and should be bolstered by openness and accountability [44, 45, 46].

One year into the COVID‐19 pandemic, much has been learned, but much still remains to be understood. Scientists, clinicians and policymakers need to convey the importance of caution in interpreting research and clinical guidance as we learn more about the pathophysiology and long‐term consequences of SARS‐CoV‐2 infection. The knowledge of viral spread and the importance of masks and physical distancing have evolved with data [47]. The same can be said for vaccine efficacy with viral variants and potential treatment options. For example, despite initial enthusiasm, hydroxychloroquine turned out to be ineffective, and unchecked dissemination of poor‐quality evidence caused harm to patients and trust in science. Dexamethasone emerged as potentially useful in decreasing mortality in severely ill, hospitalized patients [48], whereas remdesivir gave initial hope in reducing the length of hospitalization but may not impact mortality [49, 50]. Unfortunately, the limitations of trial designs are not always adequately conveyed to lay audiences by media anxious to report definitive progress, leading some to distrust the scientific process. That said, rolling out highly efficacious vaccines within one year is a scientific accomplishment previously unthinkable. Nevertheless, when people hesitate to engage with prevention or treatment measures, without firm belief in their efficacy, adherence suffers and the public health impact is undermined, perpetuating a destructive self‐fulfilling prophecy. Sound bites with exaggerated claims must be avoided, and researchers need long‐term engagement with communities to earn their trust.

## 6 Vaccination: challenges to implementation

While HIV vaccines remain elusive, several effective SARS‐coV‐2 vaccines have been developed at “warp speed” [51, 52, 53]. Although incredibly valuable, vaccines are not a silver bullet solution, and concern for emerging viral variants, or other viruses entirely, gives us pause in relying too heavily on them. For HIV, we have long heard the need to practice “safer sex” (i.e. sex with a condom or PrEP) “until there is a vaccine.” Similarly, we heard that we would need to wear masks and physical distance “until there is a vaccine” for COVID‐19. Yet, there are costs to framing the adoption of prevention strategies as time‐limited. The number of immunized individuals needed to achieve protection through herd immunity is enormous, and the rollout to date has been inadequate and inequitable globally [54]. Although trends are encouraging in some settings like Israel and the United States, the recent surge in infections in India reminds us that public health measures, like masks and physical distancing, remain critical tools. We will also need to mobilize a Herculean public health effort to administer the vaccine to billions globally, overcoming reluctance to be immunized. Socio‐behavioural science is needed to develop approaches to promote uptake of vaccines and other public health measures to control the pandemic.

The unprecedented collective effort across institutions and organizations in vaccine development, coupled with adequate funding, allows good science to progress expeditiously. However, this accelerated pace with inadequate communication about the process raised concern and increased uncertainty about vaccine safety [55]. Fear around safety and politicized uncertainty have made the dissemination of clear, comprehensible information more critical than ever. Individuals, families and communities need consistent, ongoing messaging to move from pre‐contemplation to action [56] in their engagement with clinical trial participation and vaccine adoption [57]. Although consistency may be challenging given the rapid evolution of COVID‐19 related scientific knowledge, much can be accomplished through messaging that is delivered from a vantage point of honesty and humility about the need to act on the best information available over time. Efforts to manage this tension can be seen with HIV vaccine trials in which investigators are working carefully with communities to explain the process with openness and using lay terminology [58].

Notably, most volunteers in SARS‐CoV‐2 vaccine trials have been at increased risk of exposure because of their residential and work environments. Many, if infected, face heighted risk of negative health outcomes and mortality because of pre‐existing conditions and older age [59]. Racial and ethnic minority communities are overrepresented in essential, often low‐wage service industries with increased exposure to infection and frequently live in segregated, dense settings. Because of prior research and medical trauma, these same communities often mistrust and have a tenuous relationship with the healthcare system [60]. Community‐based participatory research approaches, as has been emphasized in recent years for HIV research [61], are essential for building trust and ensuring that clinical trial enrolment and vaccine distribution are equitable and just.

## 7 Conclusions

**Figure 3:**
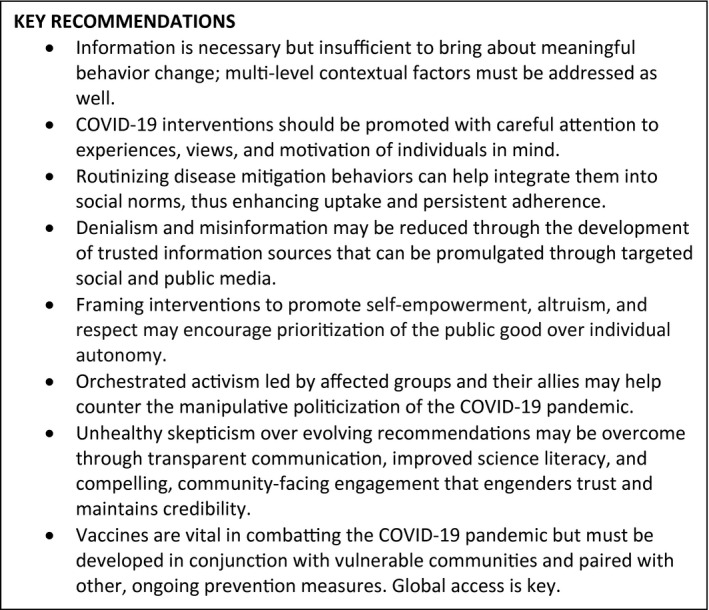
Key recommendations for combatting the COVID‐19 pandemic with socio‐behavioral science.

The COVID‐19 pandemic has wreaked havoc on the globe. As with HIV, effective tools are available to control it, but these tools require large‐scale human action to work. Understanding the social and structural context and determinants of COVID‐19 prevention behaviours through socio‐behavioural science models and theories of health behaviour change can facilitate public health efforts. Key lessons from four decades of HIV socio‐behavioural science are summarized in Figure 3. We have learned that accurate and consistent information serves as the foundation to any messaging or public health campaigns to influence individual behaviours and must be managed carefully in light of evolving evidence. Such campaigns will need to consider behavioural health, social and economic disparities, as well as cultural norms and beliefs. Manipulative politicization of effective prevention and treatment tools, however, may squander their potential impact and cripple the control the pandemic by undermining the evidence behind them and must be met with orchestrated activism. Moreover, vaccines are a critical tool, but human behaviour plays a vital role in vaccine uptake and ultimate effectiveness, and other prevention efforts must continue alongside them. Social and behavioural science is not a single, monolithic framework, and a range of perspectives within these disciplines can inform public health responses to pandemics. We need to work together to address the stigma, inequities, access to care and other societal factors that overlay individual behaviour and thus prevent us from effectively combatting the COVID‐19 pandemic. COVID‐19 has affected us all in profound ways; acknowledging and embracing the needs of individuals and the way they engage with and are affected by their communities and society will be central to ending the pandemic.
